# Changes of Phosphatidylcholine and Fatty Acids in Germ Cells during Testicular Maturation in Three Developmental Male Morphotypes of *Macrobrachium rosenbergii* Revealed by Imaging Mass Spectrometry

**DOI:** 10.1371/journal.pone.0120412

**Published:** 2015-03-17

**Authors:** Tanapan Siangcham, Piyachat Chansela, Takahiro Hayasaka, Noritaka Masaki, Morakot Sroyraya, Jaruwan Poljaroen, Saowaros Suwansa-ard, Attakorn Engsusophon, Peter J. Hanna, Prasert Sobhon, Mitsutoshi Setou

**Affiliations:** 1 Department of Anatomy, Faculty of Science, Mahidol University, Bangkok, Thailand; 2 Department of Anatomy, Phramongkutklao College of Medicine, Bangkok, Thailand; 3 Health Innovation and Technology Center, Faculty of Health Sciences, Hokkaido University, Hokkaido, Japan; 4 Department of Cell Biology and Anatomy, Hamamatsu University School of Medicine, Shizuoka, Japan; 5 Mahidol University, Nakhonsawan Campus, Nakhonsawan, Thailand; 6 Pro Vice-Chancellor’s Office, Faculty of Science, Engineering and Built Environment, Deakin University, Geelong, Australia; National Cancer Institute, UNITED STATES

## Abstract

Testis maturation, germ cell development and function of sperm, are related to lipid composition. Phosphatidylcholines (PCs) play a key role in the structure and function of testes. As well, increases of polyunsaturated fatty acids (PUFA) and highly unsaturated fatty acids (HUFA), especially arachidonic acid (ARA), eicosapentaenoic acid (EPA), and docosahexaenoic acid (DHA) are essential for male fertility. This study is the first report to show the composition and distribution of PCs and total fatty acids (FAs) in three groups of seminiferous tubules (STs) classified by cellular associations [i.e., A (STs with mostly early germ cells), B (STs with mostly spermatids), and C (STs with spermatozoa)], in three morphotypes of *Macrobrachium rosenbergii*, [i.e., small male (SM), orange claw male (OC), and blue claw male (BC)]. Thin layer chromatography exhibited levels of PCs reaching maxima in STs of group B. Imaging mass spectrometry showed remarkably high signals corresponding to PC (16:0/18:1), PC (18:0/18:2), PC (18:2/20:5), and PC (16:0/22:6) in STs of groups A and B. Moreover, most signals were detected in the early developing cells and the intertubular area, but not at the area containing spermatozoa. Finally, gas chromatography-mass spectrometry indicated that the major FAs present in the testes were composed of 14:0, 16:0, 17:0, 18:0, 16:1, 18:1, 18:2, 20:1, 20:2, 20:4, 20:5, and 22:6. The testes of OC contained the greatest amounts of these FAs while the testes of BC contained the least amounts of these FAs, and there was more EPA (20:5) in the testes of SM and OC than those in the BC. The increasing amounts of FAs in the SM and OC indicate that they are important for spermatogenesis and spermiogenesis. This knowledge will be useful in formulating diets containing PUFA and HUFA for prawn broodstocks in order to improve testis development, and lead to increased male fecundity.

## Introduction

*Macrobrachium rosenbergii*, the giant freshwater prawn, is one of the most economically important species for global freshwater prawn farming [1–2]. Knowledge of this species, especially in nutrition and reproduction, has been acquired but remains incomplete. Consequently, prawn farmers usually face many problems during culture of the animals, imbalanced lipid consumption is a common cause of low fecundity of males in many broodstock species [3–6].

In crustacean females, there are a number of reports on lipid profiles in the ovaries, and these have been used as key knowledge to formulate balanced lipid diets. For example, ovarian lipid compositions, especially triacylglycerols (TAGs) and phospholipids (PLs), have been determined for *Serolis pagenstecheri* [7], *Serolis cornuta* [7], Penaeus monodon [8], *Penaeus semisulcatus* [9], *M*. *rosenbergii* [10], *Litopenaeus* v*annamei* [11], *Fenneropenaeus indicus* [12], *Cherax quadricarinatus* [13], *Portunus sanguinolentus* [14], *Albunea symmysta* [15], and *Penaeus merguiensis* [16], and indicated that lipid changes are associated with ovarian maturation and embryonic development. This has provided data for formulated balanced lipid diets for females. On the other hand, studies in males have focused on testicular lipids, including TAGs and PLs, in *S*. *pagenstecheri* [7], *S*. *cornuta* [7], Pleoticus muelleri [17], *P*. *monodon* [8], and Macrobrachium nipponense [18]. These reports indicated that the amount of lipids in the testes were lower than the ovaries and usually contained eicosapentaenoic acid (EPA) and docosahexaenoic acid (DHA). However, arachidonic acid (ARA) was found to be higher than EPA and DHA in the spermatophores of P. monodon [3]. A knowledge of lipid composition in the testes of developing males of *M*. *rosenbergii* is now needed in order to formulate balanced diets for the improvement of male fecundity.

The PLs, especially phosphatidylcholines (PCs), are major integral components of plasma membranes, and are also involved in sperm membrane permeability and fluidity [19–22], acrosomal reactions [23], and sperm motility [24]. PCs are composed of a choline head group, glycerol, and two fatty acid side chains that can be saturated and/or unsaturated. PC treatments have prevented lipid peroxidation or degradation of enzymes in stored semen of the turkey [25], and improved acrosomal responses in human sperm [23].

It has been reported that fatty acid (FA) side chains of lipid molecules, especially in polyunsaturated fatty acids (PUFA) and highly unsaturated fatty acids (HUFA) play important roles in reproduction [4], [21], [26–30]. The three best known HUFA molecules concerned with reproduction are ARA, EPA, and DHA. ARA is a precursor of series II prostaglandins (PGs), whereas EPA is a precursor of series III PGs [31]. Both PGs are involved in steroid production [32]. The role of these two molecules and DHA has been studied in the goldfish [32], and it was found that they all control steroidogenesis in the testis, and that EPA deficiency delayed spermiation and decreased fertilization rates. For penaeid shrimps, including *P*. *monodon* and *L*. *vannamei*, it was found that the diet containing polychaetes, mollusk, squids, fish, vegetable oils which are rich in HUFA and PUFA, especially ARA, EPA, and DHA, could improve the quality of spermatophores and sperm [3, 5, 33–34]. Similarly, diet containing these natural components could also enhance male reproductive performance in *M*. *malcolmsonii* [35]. Another study reported that the EPA-containing diet enhanced sperm production in the freshwater crayfish, *Astacus leptodactylus* [4], and HUFA was found to increase the recovery of spermatogenesis in n-3 desaturase-null mice that cannot synthesize HUFA [30].

Mammalian spermatogenesis occurs in the seminiferous tubules (STs) following puberty, which starts from mitotic divisions of type B spermatogonia into primary spermatocytes [36]. The primary spermatocytes then go through meiosis I to produce secondary spermatocytes, meiosis II to produce haploid spermatids, and transformation of spermatids into spermatozoa that contain less cytoplasm [36]. Furthermore, germ cells in STs are supported by Sertoli cells or nurse cells [36–37]. So, each mammalian ST contains a mixture of developing germ cells and spermatozoa designated as cellular association, which can be classified into 14 stages in human [38].

In contrast, the STs of *M*. *rosenbergii* have been characterized into 9 maturation stages [i.e., stages I to IX], according to cellular association [39]. Stages I to V contained mostly primary and secondary spermatocytes; Stages VI to VIII contained mostly spermatids (early, middle, and late spermatids); and Stage IX contained mostly spermatozoa with decondensed chromatin. In all stages, the nurse cells and spermatogonia were always located on the basement membrane [39]. Moreover, *M*. *rosenbergii* males have been characterized into three distinct developmental morphotypes [i.e., small male (SM), orange claw male (OC), and blue claw male (BC) with fully mature testis] [40–41]. The lipids and FAs required for maturation of the STs within the three developmental male morphotypes of *M*. *rosenbergii* have not been studied. Since each ST is too small to be analysed for lipid profiles by imaging mass spectrometry (IMS), the STs were, therefore, sub-grouped into three broad maturation groups based on cellular components [i.e., A (Stages I-V), B (Stage VI-VIII), and C (only Stage IX)].

There are several ways to reveal lipid and FA compositions, namely thin layer chromatography (TLC) and gas chromatography-mass spectrometry (GC-MS). However, these methods are not able to localize the lipid molecules in tissue sections. IMS is a new technique used to determine the distribution of all lipids contained within tissues at high resolution. Recently, our collaborative research using IMS has successfully visualized seminolipid and metabolites in mouse testes [42–43], and PCs and TAGs in ovaries of *P*. *merguiensis* [16]. However, there have been no IMS analyses of male *M*. *rosenbergii* testes.

In this study, we focused on the localization and quantification of PCs, and the composition of FAs, including PUFA and HUFA, in the testes of three developmental male morphotypes of *M*. *rosenbergii*, and in during three phases of ST maturation which contain different stages of developing germ cells. The results are now being used to produce balanced formula diets for male broodstocks, especially with appropriate contents of PUFAs and HUFAs in order to increase male fecundity.

## Materials and Methods

### Animals and histology of the seminiferous tubules

**Fig 1:**
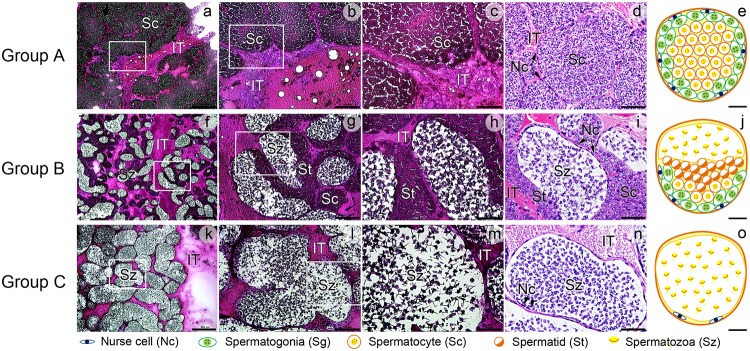
Micrographs of cryosections (a-c, f-h, k-m), H&E-stained paraffin sections (d, i, n), and illustrations of the three groups of seminiferous tubules (STs) in OC males (e, j, o). Group A STs (a-e) contain mostly spermatogonia (Sg) and nurse cells (Nc) close to the basement membrane, and few spermatocytes (Sc) in the lumen. Group B STs (f-j) contain Sg, Nc, numerous spermatids (St), and immature spermatozoa (Sz) within a lightly stained lumen. Group C STs (k-o) contain mostly fully mature Sz (with decondensed chromatin) and Nc close to the basement membrane. All stages of the STs are surrounded by intertubular areas (IT). Panels b, g, l are the higher magnifications of the boxed areas in panels a, f, k, and panels c, h, m are the higher magnifications of the boxed areas in panels b, g, l, respectively, compared with panels d, i, n which are the pictures taken from ~5μm thick H&E stained paraffin sections and the summary diagrams illustrating the characteristics of the three groups of the STs in panels e, j, o. Scale bars: a, f, k = 400 μm; b, g, l = 100 μm; c-d, h-i, m-n = 50 μm; e, j, o = 20 μm.

Thirty male giant freshwater prawns in each developmental morphotype, namely SM, OC, and BC, were obtained from a commercial farm in Suphanburi province, Thailand. The prawns were anesthetized on ice for 2 min. The testes were dissected out, (i) frozen immediately in liquid nitrogen and stored at -80°C, and (ii) fixed in 4% paraformaldehyde in 0.1 M phosphate-buffered saline (PBS; 0.033 M NaH_2_PO_4_·2H_2_O, 0.067 M Na_2_HPO_4_·H_2_O, and 0.145 M NaCl), pH 7.4, to confirm the structure by paraffin section (5 μm). The frozen testes of the three male morphotypes were divided into two equal parts for cryosection and lipid extraction S1 Fig. One part of each frozen tissue (at the base only) was attached to a specimen plate using OCT compound (Optimum Cutting Temperature, Sakura Finetek 4583, Sakura, Tokyo, Japan) and sectioned (~10 μm) with a cryostat, CM 1950, (Leica Microsystems, Wetzler, Germany), after which sections were transferred to silane-coated slides (Sigma-Aldrich, Missouri, USA) for characterizing the stages of seminiferous tubules by hematoxylin and eosin (H&E) staining Fig. 1 Left column. The sections were dried using a hair dryer and stained with Mayer's hematoxylin solution for 10 min, washed with tap water, counterstained with eosin, and mounted by Permount (Bio-Optica, Milan, Italy). They were then examined under a Nikon E600 light microscope (Nikon, Tokyo, Japan), and images were captured by a Nikon DXM digital camera using an ACT-1 program.

### Lipid extraction

The frozen testes of each group and stage were weighted, pulverized, and extracted with 0.1 g/ml of extraction solution (chloroform: methanol, 2:1 v/v) following the method described earlier [16, 42]. The samples were then sonicated for 10 s and stopped for 5 s, and this procedure was repeated 10–15 times using a Microson, Ultrasonic Cell Disruptor XL-2000 (Wakenyaku Co. Ltd., Kyoto, Japan). The glass tubes containing the sonicated tissues were tightly wrapped with parafilm, and then incubated overnight at room temperature. The samples were centrifuged at 3000 x*g* for 5 min to separate the tissue residues, and the solutions containing lipid were collected and transferred to new glass tubes, wrapped, and stored at -80°C until being analysed.

### Separation and quantification of lipids by thin layer chromatography (TLC)

The extracted lipids were separated with TLC using the method described earlier by our group [16, 42]. The solution containing extracted lipids (3 μl per sample) and the PC standard (Sigma-Aldrich, Missouri, USA) were spotted (with each spot being 5 x 1 mm in size) onto high performance thin layer chromatography glass plates (HPTLC silica gel 60 with the size 100 x 100 mm-Merck, Darmstadt, Germany), and dried at room temperature. Each HPTLC plate was immersed in a TLC chamber containing separation buffer (methylacetate, n-propanol, chloroform, and 0.25% KCl in the ratio 25:25:10:9 v/v/v/v). Each HPTLC plate was air-dried after separation, and then was sprayed with primuline reagent (Nacalai Tesque, Inc., Kyoto, Japan) composed of 1 mg of primuline in 100 ml of 80% acetone in water. After drying, the PC bands were visualized and photographed under UV light (FAS-III, Toyobo Co. Ltds, Osaka, Japan). The intensities of the bands were analysed by ImageJ software (http://rsbweb.nih.gov/ij/).

### Identification of lipids by tandem mass spectrometry (MS/MS)

The extracted lipids from testicular tissues of each maturation group of STs and male morphotypes were thoroughly mixed 1:1 v/v with matrix solution (20 mg/ml DHB in 70% methanol and 0.1% TFA). Aliquots of 1 μl of the solutions were applied manually to a stainless plate and cool air-dried using a hair dryer. A calibration process was performed using 10 pmol/μl bradykinin and 10 pmol/μl human angiotensin-II as standard peptides. The MS/MS analyses were performed using a QSTAR Elite high-performance, hybrid quadrupole TOF mass spectrometer (Applied Biosystems/MSD Sciex, Foster City, CA). The extracted lipids were ionized in positive ion mode and fragmented with collision energy between 30–40 V. After being analysed, the precursor ions were identified based on neutral losses in the product ion spectra and confirmed by using Metabolite MS Search (http://www.hmdb.ca/spectra/ms/search).

### Distributions of phosphatidylcholine by imaging mass spectrometry (IMS)

A part of each frozen testis (used for histology) was sectioned at 10 μm of thickness with a cryostat (CM 1950, Leica Microsystems). The sections were thaw-mounted onto indium tin oxide (ITO)-coated slides (Bruker Deltonics, Bremen, Germany), dried and then kept at -30°C until IMS analysis. Before IMS analyses, the sections were dried at room temperature and then sprayed with matrix solution using a 0.2-mm nozzle caliber airbrush (Procon Boy FWA Platinum, Tokyo, Japan). The matrix used was 2,5-dihydroxybenzoic acid (DHB) (Bruker Daltonics), and it was firstly dissolved to reach a concentration of 50 mg/ml in 70% methanol and 0.1% trifluoroacetic acid (TFA). A calibration process was performed using 10 pmol/μl bradykinin and 10 pmol/μl human angiotensin-II as standard peptides by applying on to the sprayed area out of the tissue sections. The sprayed sections were then analysed in a positive ion mode using an ultraflex II MALDI TOF/TOF mas nics). The mass spectra were obtained in the mass ranges between *m/z* 500–1000. The settings of laser s spectrometer (Bruker Delto irradiation were 200 Hz frequency and a raster width at 20 μm. After IMS analyses, ion images were obtained using flexImaging 2.1 software (Bruker Daltonics). Finally, the analysed sections were stained with H&E to confirm the histology of the area of interest.

### Analyses of fatty acids by gas chromatography-mass spectrometry (GC-MS)

These analyses followed the methods described earlier [16]. The extracted lipids were spiked with an internal control (0.4 mg/ml arachidic acid (20:0) diluted in chloroform:methanol at a ratio of 2:1), and then dried by nitrogen gas using a TurboVap LV Evaporation System (Caliper Life Sciences, Hopkinton, MA, USA). After being completely dried, the lipids were methylated using a fatty acid methylation kit (Nacalai Tesque, Inc., Kyoto, Japan), and then purified using a fatty acid methyl ester purification kit (Nacalai Tesque, Inc.). The purified FAs were stored at -3°C until analysed by GC-MS.

The purified FAs from testes of each morphotype were separately injected (1 μl per sample) into a GC-MS QP-2010 Plus (Shimadzu Co., Kyoto, Japan), applied with a DB-5MS column (3060.25 mm I.D., 0.25 mm; D.F., Agilent technologies, CA, USA). The purified FAs were analysed under a column temperature of 210°C and column pressure between 110 kPa-380 kPa at 7 kPa/min. After analyses, the FAs were identified and the amount calculated using the internal controls as a reference.

### Statistical analyses

The intensity of each band from TLC analyses and FAs amount of each testis stage and male morphotype from GC-MS analyses were expressed as a mean ± S.D. and the data was then compared using a Student’s *t*-test to determine differences. A probability value of less than 0.05 (*P*<0.05) indicated a significant difference.

## Results

### Histology of the seminiferous tubules

Spermatogenesis within the STs has been classified into 9 stages corresponding to the presence of different types of spermatocytes, spermatids, and spermatozoa [39]. In our research using 10 μm-thick cryosection, it was difficult to clearly identify all 9 stages of the STs. However, based on the histological outlines and abundance of spermatogonia (Sg), spermatocytes (Sc), spermatids (St), and spermatozoa (Sz) present in the tubules we could identify the stages of the STs and separated them into three groups representing early, middle, and late stages of spermatogenesis Fig. 1a-e, f-j, k-o. Group A (including stages I-V), contained Sg and nurse cells (Nc) that were located on the basement membrane, and mostly Sc Fig. 1a-e. Group B (including stages VI-VIII), contained some Sg and Sc, but mostly St and immature Sz with condensed chromatin Fig. 1f-j. Group C (stage IX), contained mostly mature Sz with de-condensed chromatin and NC, which were located close to the basement membrane Fig. 1k-o. In all stages, the STs were surrounded by intertubular area (IT) made up mainly by connective tissues. All three groups of ST stages were found in the three male morphotypes, but in different proportions. For example, SM contained mostly group A, OC contained mostly group B, and BC contained mostly group C.

### Quantification of lipids by thin layer chromatography (TLC)

**Fig 2:**
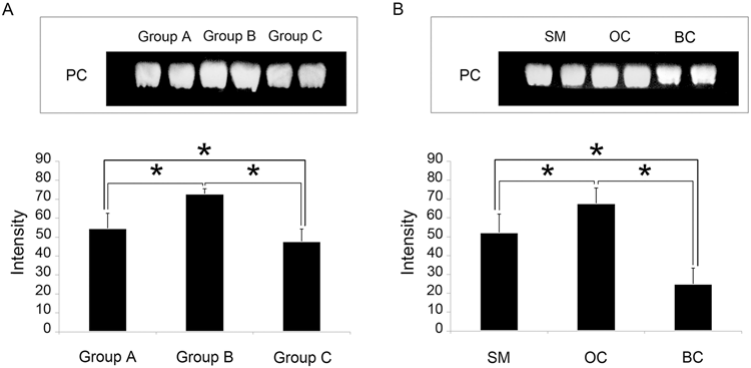
Separation and identification of phosphatidylcholines (PCs) by thin-layer chromatography (TLC) showing bands duplicated bands (upper panels) and histograms of the intensity of PCs (lower panel) in (A) each group of seminiferous tubules (ST), and (B) testes of the three developmental male morphotypes. Group B STs, containing mostly spermatids and some immature spermatozoa, show significant differences of PCs intensities compared with groups A and C (*P*<0.05; means ± S.D.; n = 5). The data support the IMS results of the PC (16:0/18:1). Moreover, the testes of OC males also contain significantly more PCs than those of SM and BC males (*P*<0.05). SM = small male; OC = orange claw male; BC = blue claw male. Bar = S.D.; * = significant difference at *P*<0.05.

The extracted lipids were separated by TLC, and the highest intensity signals were found in PC bands of each group. The PCs bands were expressed as mean ± S.D. which showed different amounts in each of the ST groups Fig. 2A and the male developmental morphotypes Fig. 2B. The STs of group B which contained mostly spermatids and some immature sperms showed significantly higher intensities compared with group A and C (*P*<0.05) Fig. 2A. The highest amounts of PCs could be observed in the OC males, which is the transitional stage from SM to BC male, and the lowest PC amounts were observed in BC males (with significant difference at *P<*0.05) Fig. 2B.

### Identification of lipids by tandem mass spectrometry (MS/MS)

**Fig 3:**
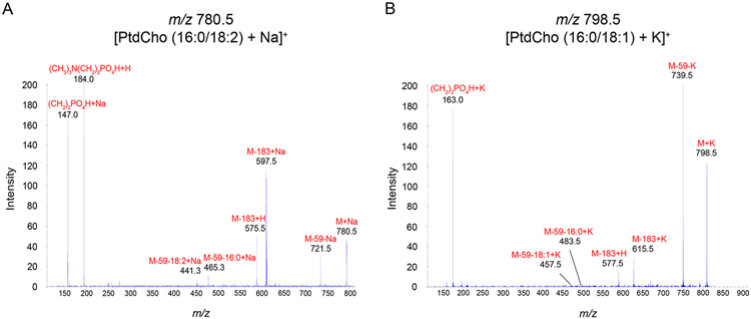
MS/MS analysis showing product ions from the precursor ions at (A) *m/z* 780.5 and (B) *m/z* 798.5. The product ions from the precursor ion at *m/z* 780.5 represent neutral losses of a PC head group [(CH_3_)_3_N(CH_2_)_2_PO_4_H] at *m/z* 597.5 and trimethylamine [(CH_3_)_3_N] at *m/z* 721.5. The minor peaks at *m/z* 465.3 and 441.3 correspond to neutral losses of FAs (16:0 and 18:2) from a peak at *m/z* 721.5. Therefore, the molecule was assigned as [PC (16:0/18:2) + Na]^+^. The product ions from the precursor ion at *m/z* 798.5 represent neutral losses of the PC head group [(CH_2_)_2_PO_4_H] at *m/z* 615.5 and trimethylamine [(CH_3_)_3_N] at *m/z* 739.5. The minor peaks at *m/z* 483.5 and 457.5 correspond to neutral losses of FAs (16:0 and 18:1) from a peak at *m/z* 739.5. Therefore, the molecule was assigned as [PC (16:0/18:1) + K]^+^.

MS/MS analysis showed product ions from precursor ions at *m/z* 780.5 Fig. 3A, and *m/z* 798.5 Fig. 3B. These signals were identified as [PC (16:0/18:2) + Na]^+^ and [PC (16:0/18:1) + K]^+^, respectively. The product ions from the precursor ion at *m/z* 780.5 represented neutral losses of a PC head group [(CH_3_)_3_N(CH_2_)_2_PO_4_H] at *m/z* 597.5 and trimethylamine [(CH_3_)_3_N] at *m/z* 721.5. These neutral losses are common for PCs. Another peak at *m/z* 575.5 indicates the replacement of adduct ion from Na^+^ to H^+^. The minor peaks at *m/z* 465.3 and 441.3 correspond to neutral losses of FAs (16:0 and 18:2) from a peak at *m/z* 721.5. Therefore, these molecules were assigned as [PC (16:0/18:2) + Na]^+^ Fig. 3A. The product ions from the precursor ion at *m/z* 798.5 represents neutral losses of a PC head group [(CH_2_)_2_PO_4_H] at *m/z* 615.5 and trimethylamine [(CH_3_)_3_N] at *m/z* 739.5. The peak at *m/z* 577.5 indicates the replacement of adduct ion from K^+^ to H^+^. The minor peaks at *m/z* 483.5 and 457.5 correspond to neutral losses of FAs (16:0 and 18:1) from a peak at *m/z* 739.5. Therefore, these molecules were assigned as [PC (16:0/18:1) + K]^+^ Fig. 3B.

All signals from ion images Table 1 were identified in the same way as the two signals described above, and comprised of *m/z* 756.5 [PC (16:0/16:1) + Na]^+^, 760.5 [PC (16:0/18:1) + H]^+^, 782.5 [PC (16:0/18:1) + Na]^+^, 798.5 [PC (16:0/18:1) + K]^+^, 780.5 [PC (16:0/18:2) + Na]^+^, 796.5 [PC (16:0/18:2) + K]^+^, 784.5 [PC (16:0/18:0) + Na]^+^, 800.5 [PC (16:0/18:0) + K]^+^, 804.5 [PC (18:2/18:2) + Na]^+^, 806.5 [PC (18:1/18:2) + Na]^+^, 808.5 [PC (18:0/18:2) + Na]^+^, 824.5 [PC (18:0/18:2) + K]^+^, and 810.5 [PC (18:0/18:1) + Na]^+^. The signals that represented omega-3 FAs Table 2 were 826.5 [PC (18:2/20:5 (EPA)) + Na]^+^, 846.5 [PC (18:0/20:5 (EPA)) + K]^+^, 828.5 [PC (16:0/22:6 (DHA)) + Na]^+^, 844.5 [PC (16:0/22:6 (DHA)) + K]^+^, 870.5 [PC (18:1/22:6 (DHA)) + K]^+^, and 872.5 [PC (18:0/22:6 (DHA)) + K]^+^, and the signals that represented omega-6 were 820.5 [PC (16:0/20:4 (ARA)) + K]^+^, 830.5 [PC (18:1/20:4 (ARA)) + Na]^+^, and 832.5 [PC (18:0/20:4 (ARA)) + Na]^+^.”

**Table 1:** MS/MS and IMS identifications and distributions of phosphatidylcholines (PCs) in three groups of seminiferous tubules (STs).

*m/z*	FA composition	Adduct	Distribution in ST groups
**756.5**	16:0/16:1	Na	--, B, C
**760.5**	16:0/18:1	H	A, B, C
**780.5**	16:0/18:2	Na	A, B, C
**782.5**	16:0/18:1	Na	A, B, C
**784.5**	16:0/18:0	Na	A, B,--
**796.5**	16:0/18:2	K	A, B, C
**798.5**	16:0/18:1	K	A, B, C
**800.5**	16:0/18:0	K	A, B,--
**804.5**	18:2/18:2	Na	A, B, C
**806.5**	18:1/18:2	Na	--, B, C
**808.5**	18:0/18:2	Na	A, B,--
**810.5**	18:0/18:1	Na	--, B, C
**824.5**	18:0/18:2	K	A, B,--

^a^--, not detected.

**Table 2:** Identification of phosphatidylcholines (PCs), containing arachidonic acid (ARA), eicosapentaenoic (EPA), and docosahexaenoic acid (DHA), and their distributions in the three groups of seminiferous tubules (STs).

*m/z*	FA composition	Adduct	Distribution in ST groups
**820.5**	16:0/20:4 **ARA**	K	A, B,--
**826.5**	18:2/20:5 **EPA**	Na	A, B,--
**828.5**	16:0/22:6 **DHA**	Na	A,--,--
**830.5**	18:1/20:4 **ARA**	Na	A, B, C
**832.5**	18:0/20:4 **ARA**	Na	A,--,--
**844.5**	16:0/22:6 **DHA**	K	A,--,--
**846.5**	18:0/20:5 **EPA**	K	A, B,--
**870.5**	18:1/22:6 **DHA**	K	A,--,--
**872.5**	18:0/22:6 **DHA**	K	A,--,--

All PC species, except those underlined in this table, were identified by MS/MS analysis, and their distributions determined by IMS. The underlined PCs were partial fragments identified by using Metabolite MS Search (http://www.hmdb.ca/labm/jsp/mlims/MSDbParent.jsp).

^a^--, not detected.

### Distributions of lipids by imaging mass spectrometry (IMS)

**Fig 4:**
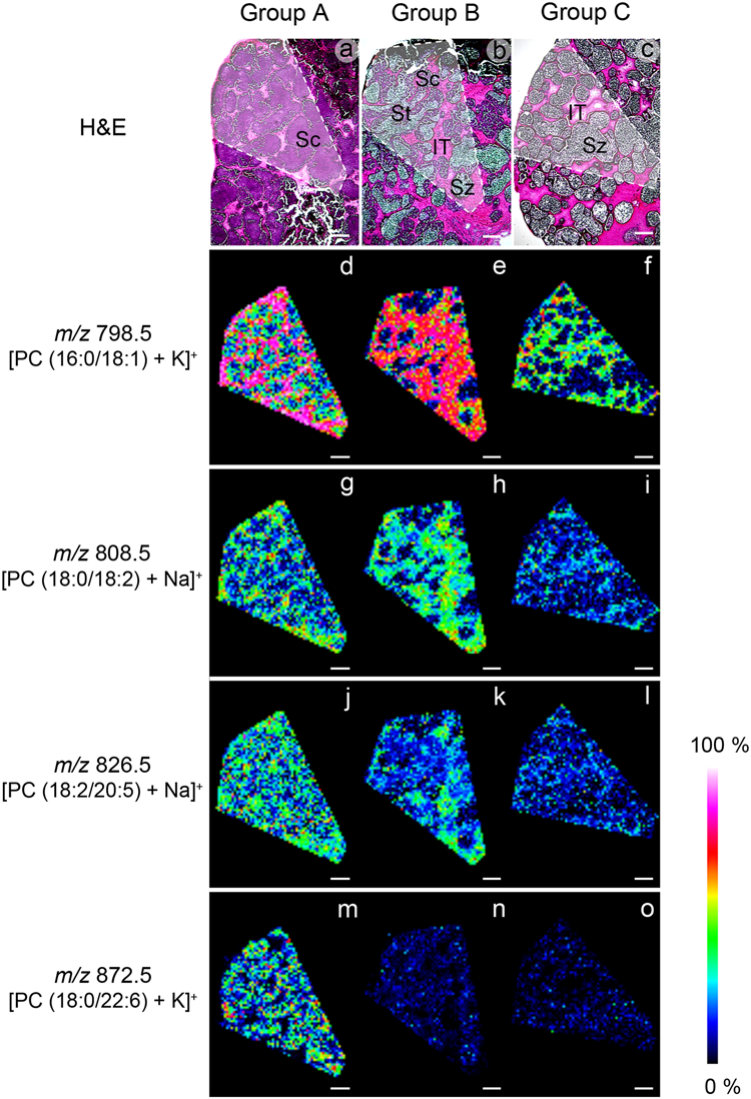
IMS showing different intensities and distributions of PCs in each group of seminiferous tubules in cryosections of the OC males (d-o), compared with picture of the same sections stained with H&E (a-c). The signals appear mainly in developing germ cells of the three groups of STs. The highest intensity corresponding to the signal at m/z 798.5, representing PC (16:0/18:1) appears in the STs of group A and B which contain mostly developing cells (d-e), and in the STs of group C which contain only mature sperms (f). The signals at m/z 808.5, representing PC (18:0/18:2 linoleic acid or LA), and *m/z* 826.5, representing PC (18:2/20:5 eicosapentaenoic acid or EPA), appear in the STs of groups A and B (g-l). The signal at *m/z* 872.5, representing PC (18:0/22:6 docosahexaenoic acid or DHA), appears in the STs of group A only (m-o). Sc = spermatocytes; Sz = spermatozoa; St = spermatids; IT = intertubular area; Scale bars = 200 μm; Relative intensity bar shows the intensity level of the ion images.

**Fig 5:**
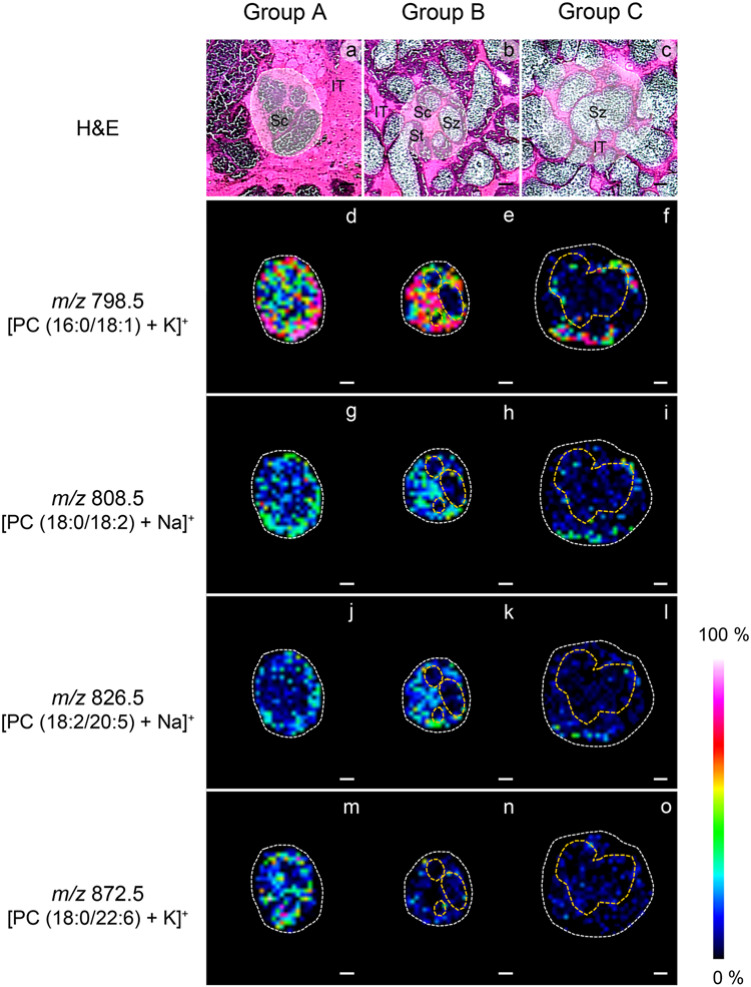
IMS showing the intensity and distribution of PCs in the STs of OC males at high magnifications. Micrographs from H&E-stained sections (a-c) show areas surrounded by white dashed lines corresponding to the same areas that display ion images (d-o). Groups A and B STs containing mostly developing cells, and intertubular area (IT) (d-e, g-h, j-k, m-n), show higher levels of the signal intensity compared with group C STs, which contains only mature spermatozoa (Sz) (surrounded by orange dashed circle) (f, i, l, o). The areas containing Sz have very low signal intensities in all groups. Sc = spermatocytes; St = spermatids; Scale bars = 200 μm; Relative intensity bar shows the intensity level of the ion images.

Ion images indicating high intensity of PCs, including *m/z* 798.5 [PC (16:0/18:1) + K]^+^, 808.5 [PC (18:0/18:2) + Na]^+^, 826.5 [PC (18:2/20:5 (EPA)) + Na]^+^, and 872.5 [PC (18:0/22:6 (DHA)) + K]^+^ in the Figs. 4 and 5, and *m/z* 756.5 [PC (16:0/16:1) + Na]^+^, 780.5 [PC (16:0/18:2) + Na]^+^, 800.5 [PC (16:0/18:0) + K]^+^, 804.5 [PC (18:2/18:2) + Na]^+^, 806.5 [PC (18:1/18:2) + Na]^+^, 810.5 [PC (18:0/18:1) + Na]^+^, 820.5 [PC (16:0/20:4 (ARA)) + K]^+^, 830.5 [PC (18:1/20:4 (ARA)) + Na]^+^, 832.5 [PC (18:0/20:4 (ARA)) + Na]^+^, 844.5 [PC (16:0/22:6 (DHA)) + K]^+^, 846.5 [PC (18:0/20:5 (EPA)) + K]^+^, and 870.5 [PC (18:1/22:6 (DHA)) + K]^+^ in the supplementary data S3 Fig., showed the distributions pattern of PCs in each ST group of OC males, particularly in groups A and B, and the IT Tables 1 and 2. The H&E-stained sections of the same areas confirmed that the identifications of the ST groups were correct S2 Fig.

The distribution of PCs were divided into 4 distinct patterns: (i) the PCs presented in all group*s* of STs comprised of PC (16:0/18:1) represented by *m/z* 760.5, 782.5, and 798.5 Figs. 4d-f and 5d-f, Table 1, PC (16:0/18:2) represented by *m/z* 780.5 and 796.5 S3b Fig. and Table 1, PC (18:2/18:2) represented by *m/z* 804.5 S3d Fig. and Table 1, and PC (18:1/20:4 (ARA)) represented by *m/z* 830.5 S3h Fig. and Table 2,which showed high signal intensities in developing germ cells area containing Sg, Sc, and St and the IT. The signal corresponding to m/z 798.5 showed the highest intensity in every group of STs Figs. 4d-f and 5 d-f, Table 1. However, we did find areas containing late St and Sz in groups B and C that showed very low intensity of *m/z* 798.5 Fig. 5e-f. (ii) The PCs presented in the STs of groups A and B, comprised of PC (16:0/18:0) represented by *m/z* 784.5 and 800.5 S3c Fig. and Table 1, PC (16:0/20:4 (ARA)) represented by *m/z* 820.5 S3g Fig. and Table 2, PC (18:2/20:5 (EPA)) represented by *m/z* 826.5 Figs. 4j-l and 5 j-l, Table 2, PC (18:0/20:5 (EPA)) represented by *m/z* 846.5 S3k Fig. and Table 2, and PC (18:0/18:2) represented by *m/z* 808.5 and 824.5 Figs. 4g-i and 5 g-i, Table 1, showed the highest signal intensities in developing germ cell areas and the IT. (iii) The PCs presented in the STs of groups B and C, comprised of PC (16:0/16:1) represented by *m/z* 756.5 S3a Fig. and Table 1, PC (18:1/18:2) represented by *m/z* 806.5 S3e Fig. and Table 1, and PC (18:0/18:1) represented by *m/z* 810.5 S3f Fig. and Table 1, also showed high signal intensities in the IT. (iv) Lastly, the PCs presented only in the STs of group A, comprised of PC (16:0/22:6 (DHA)) represented by *m/z* 828.5 and 844.5 S3j Fig. and Table 2, PC (18:0/20:4 (ARA)) represented by *m/z* 832.5 S3i Fig. and Table 2, PC (18:1/22:6 (DHA)) represented by *m/z* 870.5 S3l Fig. and Table 2, and [PC (18:0/22:6 (DHA)) represented by *m/z* 872.5 Figs. 4 m-o and 5 m-o, Table 2, showed high signal intensities in developing germ cell areas.

### Quantification of fatty acids by gas chromatography-mass spectrometry (GC-MS)

**Fig 6:**
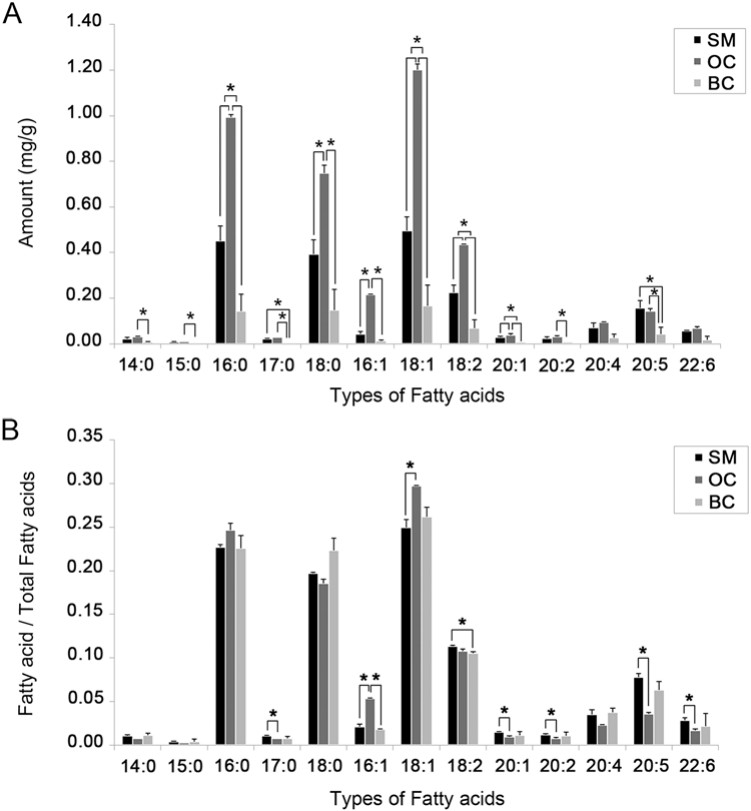
Gas Chromatography-mass spectrometry (GC-MS) analyses show (A) the FAs per testicular weight (mean ± SD; n = 5), and (B) the ratios between each FA per total FAs in the testes of the three developmental male morphotypes. (A) the FAs which were detected in the testes of the three morphotypes consisted of 14:0, 15:0, 16:0, 17:0, 18:0, 16:1, 18:1, 18:2, 20:1, 20:2, 20:4, 20:5, and 22:6. In term of relative quantities it was shown that the OC testes contained highest amounts of FAs 16:0, 18:0, 16:1, 18:1, 18:2, 20:1, and contained higher amounts of 14:0, 15:0, and 20:2 when compared with BC while the differences were not significant when compared to SM. Moreover, testes of SM and OC contained higher amounts of FAs 17:0, and 20:5 (EPA) when compared to BC, whereas FAs 20:4 (ARA) and 22:6 (DHA) showed no statistical difference among the testes of the three groups. (B) FA ratios showed that the testes of SM contained higher accumulations of 17:0, 20:1, 20:2, 20:5 (EPA) and 22:6 (DHA) when compared with OC while there were no significant differences between SM and BC. 20:4 = ARA, Arachidonic acid; 20:5 = EPA, Eicosapentaenoic acid; 22:6 = DHA, Docosahexaenoic acid; SM = small male; OC = orange claw male; BC = blue claw male; * = significant difference at P<0.05.

FAs in lipid extractions from the testes of each developmental male morphotype were quantified using GC-MS, and it was found that the FAs which were detected in the testes of the three morphotypes consisted of 14:0, 15:0, 16:0, 17:0, 18:0, 16:1, 18:1, 18:2, 20:1, 20:2, 20:4, 20:5, and 22:6. In term of relative quantities it was shown that during the development from SM to mature BC, the OC testes contained highest amounts of FAs 16:0, 18:0, 16:1, 18:1, 18:2, 20:1 (with significant difference at *P<*0.05), and contained higher amounts of 14:0, 15:0, 20:2 when compared with BC (with significant difference at *P<*0.05) while the differences were not significant when compared to SM Fig. 6A. Moreover, testes of SM and OC contained higher amounts of FAs 17:0, and 20:5 (EPA) when compared to BC (with significant difference at *P<*0.05), whereas FAs 20:4 (ARA) and 22:6 (DHA) showed no statistical difference among the testes of the three groups Fig. 6A. However, FA ratios showed that the testes of SM contained higher accumulations of 17:0, 20:1, 20:2, 20:5 (EPA) and 22:6 (DHA) when compared with OC (with significant difference at *P<*0.05) while there were no significant differences between SM and BC Fig. 6B.

## Discussion

This study, using IMS and related techniques, is the first to show the localization and composition of PCs and FAs, especially HUFA and PUFA, in three maturing ST groups of the three male morphotypes of M. rosenbergii. We focused on the changes of PCs and FAs during ST maturation, and developmental stages of male morphotypes during maturation from young SM to mature BC [39]. We found that (1) STs of group B had higher amounts of PCs and FAs than groups A and C; (2) OC males always contain higher amounts of PCs and FAs (except EPA) than the SM and BC; (3) SM males contain high ratios of HUFAs and PUFAs; (4) EPA is always higher than DHA in all ST groups, and in all male morphotypes; and (5) the PCs identified in the testes of this species were considerably higher in developing germ cells and the IT.

The composition of total lipids in each developmental morphotype of *M*. *rosenbergii* has been found to be different [44]. In particular, it was found that the total lipids in the hepatopancreas, a major energy storage organ in crustaceans, to be highest in OC males and lowest in mature BC males. This result supports our TLC results that showed trends of PC amounts in the three developmental morphotypes Fig. 2B. The OC males are reproductively less active than BC males, but are growing more rapidly than young SM and mature BC males [44–47]. Surprisingly, in OC males the group B STs with differentiating Sts had higher levels of PCs than group C STs that contain only spermatozoa Fig. 2A. It was reported that the decrease of lipid levels in the testes of BC males may relate to germ cell developmental processes in which there is an extrusion of numerous cytoplasmic components including lipids as they become mature spermatozoa [44–47]. This also supports our results as the lowest amounts of PCs and FAs were detected in the testes of BC males Figs. 2 and 6A.

IMS is a powerful technique to reveal the location of the lipids such as PCs, phosphatidylinositols, phosphatidylethanolamines, seminolipids, and TAGs in the reproductive organs of mice and shrimps, without contamination that may be introduced with embedding media [16, 42]. Recently, Goto-Inoue (2012) reported that lipids changed during testis maturation in mice, especially lipids in the positive ion mode detected in the range of *m/z* 700–900, with substantial signals corresponding to PCs. The highest intensity of these was found at m/z 798.5 [PC (16:0/18:1) + K]^+^ [43]. Moreover, Chansela (2012) reported that there were relatively large amounts of PCs in the ovary of *P*. *merguensis* [16]. Our IMS results of the testis of *M*. *rosenbergii* showed numerous signals corresponding to PCs, which included HUFA-containing PCs with m/z 820.5 [PC (16:0/20:4 (ARA)) + K]^+^, m/z 826.5 [PC (18:2/20:5 (EPA)) + Na]^+^, m/z 828.5 [PC (16:0/22:6 (DHA)) + Na]^+^, m/z 832.5 [PC (18:0/20:4 (ARA)) + Na]^+^, m/z 844.5 [PC (16:0/22:6 (DHA)) + K]^+^, m/z 846.5 [PC (18:0/20:5 (EPA)) + K]^+^, m/z 870.5 [PC (18:1/22:6 (DHA)) + K]^+^, and m/z 872.5 [PC (18:0/22:6 (DHA)) + K]^+^. These signals were present in the IT and developing germ cell areas, including Sg, Sc, and St, but not in spermatozoa Figs. 4 and 5, S3 Fig. However, the compositions of PLs in each type of developing male germ cells were found to be different. In mammals, PUFA and the major HUFA (namely DHA) accumulated at highest levels in cell membranes of male germ cells, and were essential for male fertility [48–49]. In rats, the spermatids contain more docosapentaenoic acid (DPA)-containing phospholipids than spermatocytes [50], indicating that there are species differences in the types of lipids, and qualities of developing male germ cells, implying their importance during differentiation.

Our GC-MS analysis showed the highest amount of all FAs, including 14:0, 15:0, 16:0, 17:0, 18:0, 16:1, 18:1, 18:2 (linoleic acid), 20:1, and 20:2, in the STs of OC males. Furthermore, it showed that the EPA level was higher than that of DHA in all male morphotypes, of *M*. *rosenbergii*. Notable increase of HUFA including DPA also occur during the maturation of testes in cattle [26], rats [27], hamster [51], mouse [51], guinea pig [51], dog [51], boars [28], rams [29], and monkeys [21], as this may be related to sperm high mobility facilitated by the more fluid membrane. The levels of FAs in *M*. *rosenbergii* testes were considerably lower compared with the ovaries of *P*. *merguensis* [16], and *M*. *rosenbergii* [10]. We suggest that these different levels of FAs may be related to a greater lipid requirement by oogenesis. Furthermore, the levels of HUFA in the male of this species are decreasing in the testes of the blue claw males which contained mostly mature sperm cells with small membranes of early germ cells (Sg, Sc). In SM and OC, the testes contain large amounts of developing germ cells in the spermatogenic zone (in STs of groups A, B), which is highly active in spermtogenesis [52]. After maturation, the testes of BC males contain much thinner spermatogenic zone, and mature Sz, thus the STs function is more in the storage of Sz rather than producing Sz [52]. In addition the Sz of this prawn are immobile due to the lack of tail and the nuclear chromatin is totally decondensed [39]. They are thus relatively inert compared to the mammalian sperm. It is possible that their membranes are less fluid and need much less HUFA when they reach complete maturity.

Finally, we recommend that diets containing lipids with high levels of HUFA, PUFA, especially EPA and DHA, should be given to the SM males for improving germ cell development and increase energy accumulation to shorten their developmental processes. This knowledge could be useful in formulating suitable nutrition to each male morphotype broodstock of M. rosenbergii, which is important commercial species in freshwater prawn farming countries.

## Supporting Information

S1 Fig Schematic diagram of Materials and Methods. (PDF)

S2 Fig Micrograph from H&E-stained sections showing the areas in the three ST groups being analysed in Fig. 4. The upper row (a, b, c) shows low magnification and the lower row (d, e, f) shows higher magnifications of the boxed areas. Each of the group B STs contains a narrow crescentric strip of early germ cells surrounded by red dashed lines, while the remaining part of the tubule contains spermatozoa surrounded by yellow dashed lines (b, e). In contrast, all group C STs contain only spermatozoa (surrounded by yellow dashed line) with no developing cell areas (c, f). These areas were analysed by IMS. The arrowheads indicate the laser scars that appear after IMS analyses. Sc = spermatocytes; Sz = spermatozoa; St = spermatids; IT = intertubular Scale bars; upper layer = 400 μm, lower layer = 200 μm.
(PDF)

S3 Fig Ion images show different intensities and distributions of PCs in each seminiferous group in cryosections of the OC testes, compared with H&E staining of the same areas (Top row). The signals also appear to be mainly in early germ cells and intertubular area (IT) of the three groups of STs. Sz = spermatozoa; Scale bars = 200 μm; Relative intensity bar shows the intensity level of ion images.
(PDF)
