# Supplementary figures and images for: Changes of Phosphatidylcholine and Fatty Acids in Germ Cells during Testicular Maturation in Three Developmental Male Morphotypes of Macrobrachium rosenbergii Revealed by Imaging Mass Spectrometry
Source: PLoS One. 2015 Mar 17;10(3):e0120412. doi: 10.1371/journal.pone.0120412 (PMC4363669; doi:10.1371/journal.pone.0120412)

**S1 Fig. Schematic diagram of Materials and Methods**

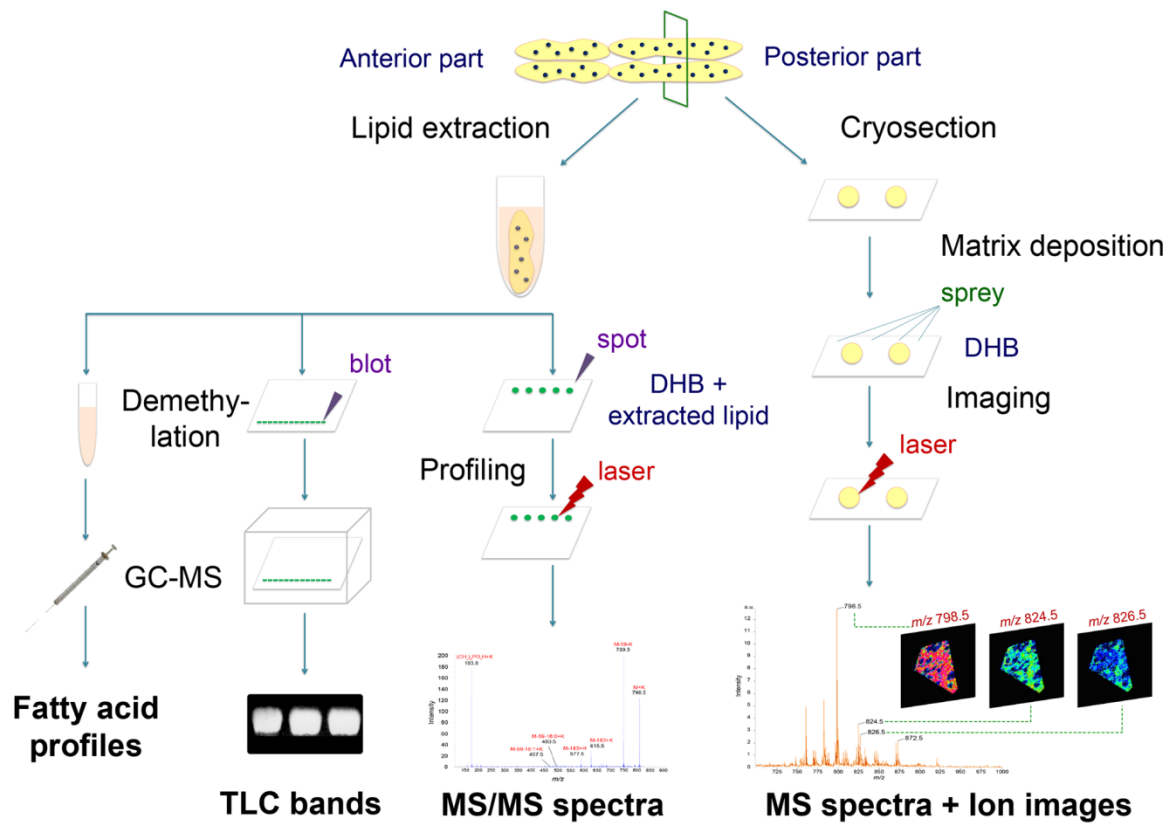

Supplement: S1 Fig — (PDF) [file pone.0120412.s001.pdf]
