# Supplementary material for: Changes of Phosphatidylcholine and Fatty Acids in Germ Cells during Testicular Maturation in Three Developmental Male Morphotypes of Macrobrachium rosenbergii Revealed by Imaging Mass Spectrometry
Source: PLoS One. 2015 Mar 17;10(3):e0120412. doi: 10.1371/journal.pone.0120412 (PMC4363669; doi:10.1371/journal.pone.0120412)

S2 Fig. Micrograph from H&E-stained sections showing the areas in the three ST groups being analysed in Figure 4.

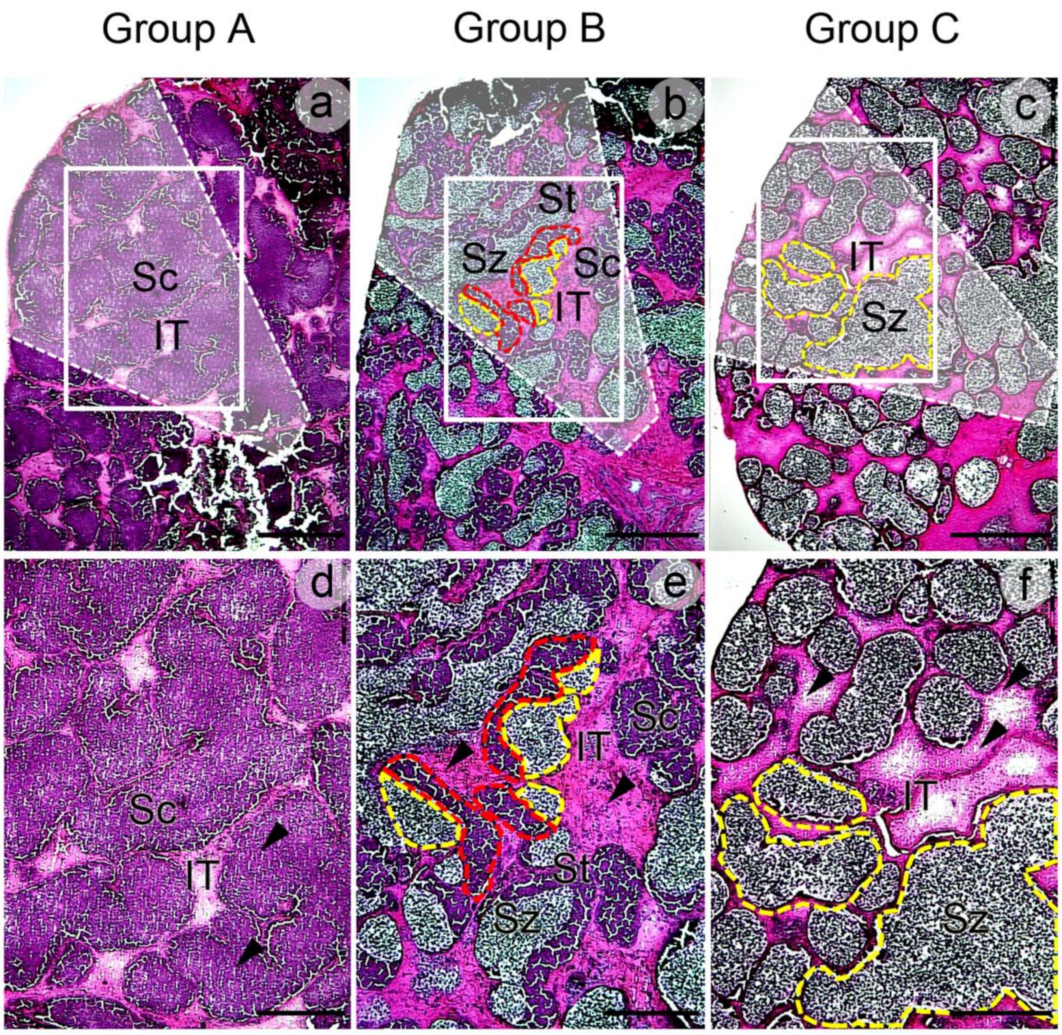

Supplement: S2 Fig — The upper row (a, b, c) shows low magnification and the lower row (d, e, f) shows higher magnifications of the boxed areas. Each of the group B STs contains a narrow crescentric strip of early germ cells surrounded by red dashed lines, while the remaining part of the tubule contains spermatozoa surrounded by yellow dashed lines (b, e). In contrast, all group C STs contain only spermatozoa (surrounded by yellow dashed line) with no developing cell areas (c, f). These areas were analysed by IMS. The arrowheads indicate the laser scars that appear after IMS analyses. Sc = spermatocytes; Sz = spermatozoa; St = spermatids; IT = intertubular Scale bars; upper layer = 400 μm, lower layer = 200 μm. (PDF) [file pone.0120412.s002.pdf]
