# Supplementary material for: Changes of Phosphatidylcholine and Fatty Acids in Germ Cells during Testicular Maturation in Three Developmental Male Morphotypes of Macrobrachium rosenbergii Revealed by Imaging Mass Spectrometry
Source: PLoS One. 2015 Mar 17;10(3):e0120412. doi: 10.1371/journal.pone.0120412 (PMC4363669; doi:10.1371/journal.pone.0120412)

S3 Fig. Ion images show different intensities and distributions of PCs in each seminiferous group in cryosections of the OC testes, compared with H&E staining of the same areas (Top row).

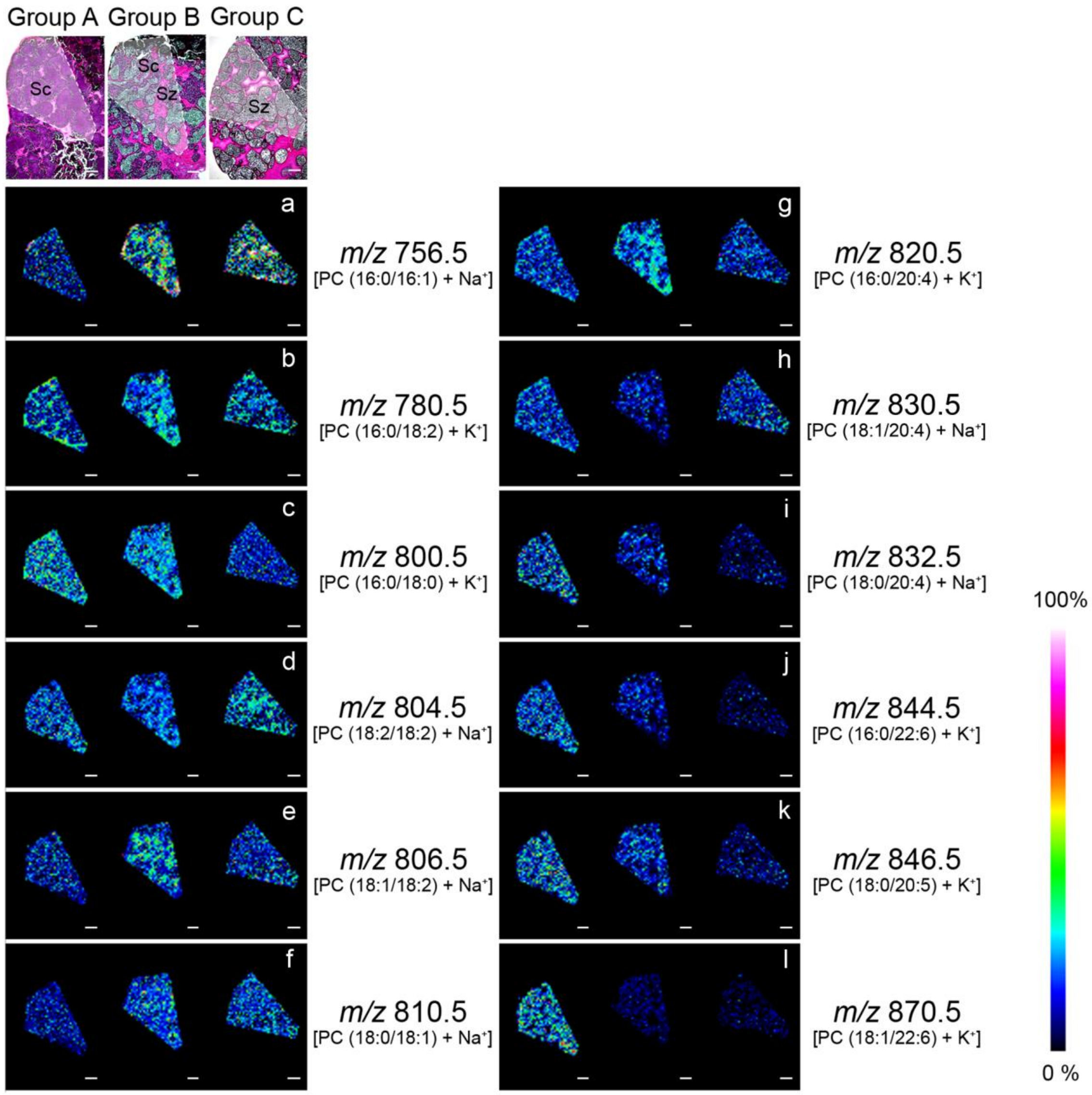

Supplement: S3 Fig — The signals also appear to be mainly in early germ cells and intertubular area (IT) of the three groups of STs. Sz = spermatozoa; Scale bars = 200 μm; Relative intensity bar shows the intensity level of ion images. (PDF) [file pone.0120412.s003.pdf]
